# The association between potential predictors and death of patients during the COVID-19 pandemic in Shiraz: a hierarchical multiple regression analysis

**DOI:** 10.1186/s12889-024-19372-2

**Published:** 2024-07-23

**Authors:** Zinat Mohebbi, Parvin Ghaemmaghami, Melika Rajaei, Mohammad Mehdi Keshtkar, Sina Ghanbarzadeh, Bagher Khoram

**Affiliations:** 1 Community Based Psychiatric Care Research Center, Department of Nursing, School of Nursing and Midwifery, Shiraz University of Medical Sciences, Shiraz, Iran; 2 School of Nursing and Midwifery, Shiraz University of Medical Sciences, Shiraz, Iran; 3 Nurse, Shiraz University of Medical Sciences, Shiraz, Iran; 4 Nurse, Fasa University of Medical Sciences, Fasa, Iran; 5 Medical Student, Student Research Committee, Medical College, Shiraz University of Medical Sciences, Shiraz, Iran; 6 Instructor of Critical-Care Nursing, MSN, Department of Anesthesia, School of Nursing and Midwifery, Shiraz University of Medical Sciences, Shiraz, Iran

**Keywords:** Clinical and para clinical manifestation, Mortality, COVID-19, Alive and dead patients, Infection

## Abstract

**Introduction:**

Identifying clinical factors that increase the risk of mortality in COVID-19 patients is crucial. This enables targeted screening, optimizing treatment, and prevention of severe complications, ultimately reducing death rates. This study aimed to develop prediction models for the death of patients (i.e., survival or death) during the COVID-19 pandemic in Shiraz, exploring the main influencing factors.

**Method:**

We conducted a retrospective cohort study using hospital-based records of 1030 individuals diagnosed with COVID-19, who were hospitalized for treatment between March 21, 2021, and March 21, 2022, in Shiraz, Iran. Variables related to the final outcome were selected based on criteria and univariate logistic regression. Hierarchical multiple logistic regression and classification and regression tree (CART) models were utilized to explore the relationships between potential influencing factors and the final outcome. Additionally, methods were employed to identify the high-risk population for increased mortality rates during COVID-19. Finally, accuracy was evaluated the performance of the models, with the area under the receiver operator characteristic curve(AUC), sensitivity, and specificity metrics.

**Results:**

In this study, 558 (54.2%) individuals infected with COVID-19 died. The final model showed that the type of medicine antiviral (OR: 11.10, *p* = 0.038) than reference (antiviral and corticosteroid), and discharge oxygen saturation(O2) (OR: 1.10, *p* < 0.001) had a positive association with the chance of survival, but other variables were not considered as predictive variables. Predictive models for the final outcome(death) achieved accuracies ranging from 81 to 87% for hierarchical multiple logistic regression and from 87 to 94% for the CART model. Therefore, the CART model performed better than the hirerical multiple logistic regression model.

**Conclusion:**

These findings firstly elucidate the incidence and associated factors of the outcome (death) among patients in Shiraz, Iran. Furthermore, we demonstrated that antiviral medication alone (without corticosteroids) and high O2 increase the survival chances of COVID patients.

**Supplementary Information:**

The online version contains supplementary material available at 10.1186/s12889-024-19372-2.

## Introduction

The spread of coronavirus disease around the world is a great challenge affecting all aspects of people’s lives. Almost all people around the world are susceptible to SARS-CoV-2 infection [1]. Coronavirus disease 2019 (COVID-19), the disease of the new century, is an acute respiratory syndrome caused by coronavirus type 2 (SARS-COV2) [2]. This virus has the ability to infect humans, bats, and some mammals; it caused one of the most devastating epidemic in 2003 and 2003. Due to the complications and mortality caused by this disease, the World Health Organization declared this disease an epidemic on 11 March 2020 [3–5]. This disease has a broad spectrum of clinical manifestations, of which fever and cough are the most common ones [6]. In 30–60% of patients with COVID-19, there is shortness of breath, which occurs on average 5–8 days after the initial infection. In addition, in patients over 60 years of age, hypoxemia may also be observed [7]. Due to the spread of this virus variants and its different symptoms, predicting the course of the disease is complicated. Although COVID-19 is recognized as a respiratory disease, due to varying degrees of disease severity, a wide range of clinical manifestations from complete asymptomatic to severe pneumonia-related death have been reported. Following the aggravation of acute respiratory distress syndrome (ARDS) symptoms and cytokine storm, multi-organ failure occurs, which causes extensive changes in laboratory data and increases the mortality rate [8–10]. Although the ratio of the number of deaths to the number of recoveries is low, as reported 1.2% in the city of Shiraz (the capital of Fars province, which is located in the southwest of Iran), the rapid transmission of the virus leads to a high incidence rate and, as a result, a high mortality rate. The mortality rate of COVID-19 was 8119 people until the time of writing this article. Therefore, it is necessary to identify the factors related to the increased risk of death/mortality rate in COVID-19 patients. These factors include old age, male gender, comorbidities (diabetes, high blood pressure, reduced lung and kidney function, and other chronic diseases), racial/ethnic disparities, and some biomarkers [11, 12]. The connection between scientific and medical communities has led to the identification of reliable biomarkers related to COVID-19 and its progress [13]. Discerning effective biomarkers, in addition to having an important role in the screening of these patients, contributes to rapid diagnosis, appropriate management of treatment options, and prevention of serious complications [14, 15]. Identification laboratory and cilinical information will be useful for predicting the disease progress, identifying prevention strategies, and thus reducing mortality. Given that so far the hierarchical logistic regression model has not been used to investigate the relationship between mortality and related factors in this pandemic. Additionally, decision trees can identifying the most influential factors for predicting an outcome and can discern and describe nonlinear relationships. Their results are presented in an intuitive flowchart format, facilitating interpretation, and they possess the ability to categorize individuals hierarchically based on various factors. Furthermore, decision trees can uncover multiple interactions without prior assumptions. While prevalence rates are typically compared across strata of only one or two independent variables, particularly in public health monitoring and reporting, classification trees enable more efficient utilization of available surveillance data by facilitating the simultaneous analysis of multiple independent variables. These attributes render decision tree methods advantageous compared to traditional regression techniques commonly used in the social and behavioral health sciences. Accordingly, use of classification trees may support a more precise identification of population groups that are heterogeneous in terms of Covid disease [16,17]. Same as hierarchical logistic regression model, small number of studies have used decision tree approaches to examine the factors associated with covid disease.

The present study used both hierarchical logistic regression and classification trees to develop a predictive model for final outcome among covid patients. Hierarchical multiple logistic regression and classification and regression tree (CART) models provide numerous advantages compared to conventional models such as logistic regression. These advantages include enhanced modeling complexity, greater flexibility in modeling, the ability to model interactions between variables, and the capability to detect and distinguish complex patterns. Utilizing hierarchical multiple logistic regression and CART models as alternatives to traditional logistic regression models can enhance the performance and accuracy of statistical analyses in research settings. At first, we used the two model to investigate the association between potential predictors with death of the patient (i.e., live and death) during the COVID-19 pandemic in Shiraz, Iran. Ultimately, the accuracy of the two models in predicting mortality is assessed and compared using appropriate metrics.

## Method

In this descriptive-analytical retrospective cohort study, patients who had positive PCR and pneumonia according to physician’s diagnosis and Chest X-ray results were considered as COVID-19 patients. The study’s data collection involved hospital-based records of individuals diagnosed with COVID-19 who were hospitalized for treatment between March 21, 2021, and March 21, 2022. The data were gathered in accordance with the standard admission protocols of healthcare facilities referring to the intensive care units (ICUs) of four hospitals using a convenience sampling method. A total of 1030 COVID-19 patients admitted to the ICUs of these hospitals were identified, with 256 patients allocated to Ali Asghar Hospital, 153 patients to Nemazee Hospital, 480 patients to Shahid Faghihi Hospital, and 141 patients to Shahid Chamran Hospital, ensuring comprehensive data capture by clinical staff. There were no specific inclusion or exclusion criteria in this study; only individuals who tested positive for COVID-19 and were hospitalized at these four centers were eligible.

The method of univariate logistic regression and decision tree model assessed the association of every potential predictor (independent variable) individually with death of the patient (outcome variable).

Demographic particulars, encompassing age, gender, and education level were garnered through a questionnaire. Also, we extracted the patients’ information from the medical files manually in 2022. Clinical characteristics included underlying diseases, history of COVID-19, length of hospitalization, type of medication, category of underlying disease, Oxygen saturation (O2), White Blood Cells count (WBC), C-Reactive Protein (CRP), Serum Glutamic Oxaloacetic Transaminase (SGOT) or AST(Aspartate Aminotransferase), Serum Glutamic Pyruvic Transaminase (SGPT) or ALT(Alanine Aminotransferase), Total bilirubin, Direct bilirubin, and Partial Pressure of Oxygen (Po2), the type of medicine, history of COVID19 and category of underlying disease) were recorded in both hospitalization and discharge time. In this study, a specific questionnaire was designed based on the assumptions, research questions, and variables under investigation.

### Statistical analysis

Descriptive statistics, such as means, medians, standard deviations (SDs) and interquartile range (IQR) for continuous variables and frequencies and percentages for categorical variables, were used for all demographic variables. The method of univariate logistic regression assessed the association of every potential predictor individually with death of the patient (outcome variable). Following the univariate analyses, variables showing significant associations with the death were included in two models. The hierarchical logistic regression analyses (method: Enter) and decision tree model were then used to assess the association of each potential predictor with the patient’s death (outcome variable).

#### Hierarchical multiple logistic regression analyses

The method of Hierarchical multiple logistic regression analyses, In the first step, the death of the patient was assessed regarding their association with significant demographic characteristics. In the second step in the regression analyses, underlying disease, O2 saturation, WBC, type of medicine, history of COVID-19, and category of the underlying disease were entered by enter method in addition to the first step CRP in both hospitalization and discharge time, SGOT, and SGPT in discharge time were entered in the three step, as we aimed to investigate whether the inclusion of these variables increased the prediction accuracy by each block of variables entered in the dependent variable (death of the patient) after controlling for the previously entered variables. In this method, adjusted odds ratios (ORs) and corresponding 95% confidence intervals (CIs) were reported.

#### Classification and regression trees (or CART)

Based on the results of the univariate logistic regression, variables related to death were selected. CART analysis was performed to identify high-risk populations for increased mortality during COVID-19 and the factors that most deeply influenced the increase in death. The CART model constructs a binary classification system (tree) via recursive partitioning, effectively dividing the dataset into increasingly homogeneous subgroups. At each node, the CART algorithm identifies the explanatory variable and splitting value that optimize discrimination between two outcome classes. A complete CART algorithm continues adding nodes until they achieve homogeneity or contain only a small number of observations (≥ 5, as per standard practice). The challenge in developing a useful tree lies in determining appropriate criteria for pruning. The overarching principle of pruning is that the optimal tree size minimizes misclassification rates for individuals not present in the original dataset [18]. In decision trees, the the relationships between various nodes in the classification tree are logically established based on whether a respondent exhibits a specific characteristic at each node or not. Ultimately, we evaluated the performance of the CART model by computing sensitivity, specificity, and AUC (Area Under Curve) All analyses were performed using the IBM SPSS Statistics for Windows, version 22.0 (IBM Corp, Armonk, NY). The statistical significance level was set at less than 0.05.

## Results

### Sample characteristics

A total of 1030 patient were investigated between March 2021 and March 2022 in this study. 558 (54.2%) individuals infected with COVID-19 died, and the rest survived.

The gender of the patients with COVID-19 was almost equal. Most of the patient’s education was diploma and lower than diploma (668, 87.4%), and the rest had university education. (96, 12.6%( Most of the patients (756, 73.4%) had underlying diseases and had no history of COVID-19 infection (658, 89.9%). The mean age and length of hospitalization of the patients with COVID-19 were 59.66 years old [standard deviation (SD) = 17.10], ranging from 3 to 100 years and 12.32 days [SD = 11.11], with a range of 1- 122 days. Detailed patients’ demographic characteristics are presented in Table 1.

**Table 1:** Demographic characteristics of study subjects (n = 1030)

Demographic characteristics^*^	N	%
Gender		
Female	465	45.1
Male	565	54.9
Education		
Lower than diploma	506	66.2
Diploma	162	21.2
University	96	12.6
Underlying disease		
Have	756	73.4
Not have	274	26.6
Type of medicine		
Antivirus	221	23.7
Corticosteroid	165	17.7
Antivirus & Corticosteroid	545	58.5
History of Covid19		
Have	74	10.1
Not have	658	89.9
Category of underlying disease		
Respiratory	273	26.5
Internal	229	22.2
Internal-respiratory	182	17.7
Not have	346	33.6

*The number of subjects may vary because of missing values

### Characteristics of laboratory indicators

The patient’s comprehensive characteristics of laboratory indicators are represented in Table 2. A total of 1030 patients were selected; all patients from this survey were recognized as the COVID-19 cases.

**Table 2:** Characteristics of laboratory indicators of study subjects (n = 1030)

Laboratory indicators	Mean	SD	Median	IQR
O2 saturation				
Hospitalization	82.78	12.84	86	78–92
Discharge	80.80	18.50	90	70–93
WBC				
Hospitalization	9.06	5.64	7.80	5.7–11.3
Discharge	12.77	7.78	10.90	7.40-16.28
CRP				
Hospitalization	61.74	30.73	64	44.7–82.0
Discharge	41.98	36.77	30.00	9.30-74.25
SGOT				
Hospitalization	77.33	203.59	52.00	37–76
Discharge	109.73	358.52	43.00	30–65
SGPT				
Hospitalization	63.93	209.10	40	27–77
Discharge	108.31	408.94	44	27–77
Total bilirubin				
Hospitalization	1.18	3.63	0.79	0.54–1.10
Discharge	1.52	4.63	0.83	0.51–1.33
Direct bilirubin				
Hospitalization	0.46	1.29	0.3	0.2–0.45
Discharge	0.74	2.54	0.3	0.2–0.53
Po2				
Hospitalization	51.42	38.53	42	30.2–58.4
Discharge	54.33	26.70	48.9	37–65

*SD: Standard Deviation, IQR: Quartile25-Quartile75

Before exploring the relationship between variables and the binary patient outcome, we conduct a thorough investigation into the selection of relevant and influential variables for model inclusion. Certain variables are chosen based on established knowledge and expert insights extracted from hospital records. This methodology integrates prior knowledge with data-driven analysis to pinpoint crucial variables. Initially, each variable undergoes individual assessment via univariate logistic regression, with those exhibiting a probability value exceeding 0.2 being incorporated into the final model.

### Univariate logistic regression models

Univariate logistic regression with the patients who died as a reference group revealed that gender, length of hospitalization, diploma category, all category of underlying disease, all Type of medicine, O2 saturation, WBC, CRP in both hospitalization and discharge time, SGOT and SGPT in discharge time were significantly associated with death characteristics status when analyzed as single predictors, while the other variables did not show a significant association. (Table 3).

**Table 3:** Univariate logistic regression analysis of the death of patients during the COVID-19

Variable		OR^*^	95%CI^*^	*P* Value
Gender		1.001	[0.783,1.281]	0.991
Age		0.965	[0.958,0.973]	*P* < 0.001
Education				
Lower than diploma		0.684	[0.438,1.069]	0.096
Diploma		0.527	[0.315,0.882]	0.015
Length of hospitalization		0.988	[0.976,0.999]	0.040
Category of underlying disease				
Not have		3.604	[2.579,5.037]	*P* < 0.001
Respiratory		2.990	[2.111,4.235]	*P* < 0.001
Internal		1.692	[1.164,2.459]	*P* < 0.001
History of covid19		1.704	[1.028,2.825]	0.039
Type of medicine				
Antiviral		2.543	[1.842,3.510]	*P* < 0.001
Corticosteroid		1.482	[1.044,2.103]	0.028
O2 saturation	Hospitalization	1.058	[1.045,1.071]	*P* < 0.001
	Discharge	1.175	[1.148,1.203]	*P* < 0.001
WBC	Hospitalization	0.970	[0.946,0.995]	0.017
	Discharge	0.831	[0.808,0.855]	*P* < 0.001
CRP	Hospitalization	0.995	[0.991,0.999]	0.020
	Discharge	0.976	[0.971,0.982]	*P* < 0.001
SGOT	Hospitalization	1.00	[0.999,1.001]	0.870
	Discharge	0.978	[0.973,0.984]	*P* < 0.001
SGPT	Hospitalization	1.00	[1.000,1.001]	0.561
	Discharge	0.996	[0.994,0.998]	*P* < 0.001
Total Bilirubin	Hospitalization	1.011	[0.975,1.049]	0.553
	Discharge	0.971	[0.924,1.021]	0.246
Direct Bilirubin	Hospitalization	0.993	[0.989,1.109]	0.898
	Discharge	0.901	[0.805,1.008]	0.069
Po2	Hospitalization	0.998	[0.994,1.002]	0.354
	Discharge	0.997	[0.991,1.002]	0.276

*Odds Ratio, CI: Confidence Interval

*Note* Reference group in regression analysis: death patient group; Gender (coding: male = 1, female = reference); Education: (coding: Lower than diploma = 1, Diploma = 2, University = reference category); Category of underlying disease: (coding: Not have = 0, Respiratory = 1, Internal = 2, Internal-respiratory = reference category); Type of medicine: (coding: antiviral = 1, corticosteroids = 2, 1,2 = reference category)

### Hierarchical multiple logistic regression analyses

The results of multiple hierarchical logistic regression analysis using patient death status as the dependent variable are shown in Table 4. This analysis was conducted in order to examine the contributions of variable blocks entered the prediction of patient death status simultaneously.

In the first step, age, education and length of hospitalization were assessed by inter method. In this block, gender variable made non-significant contributions to the patient’s death status, but age and educational level of the diploma compared to the reference and hospitalization period were significant predictors in this model. In the step 2, underlying diseases, history of COVID-19, type of medicines, O2 saturation, and WBC (in hospitalization and discharge time) were included in the model. In this block, age, educational level of the diploma compared to the reference and hospitalization period, type of medicine of the antiviral compared to the reference, O2 saturation and WBC in discharge were significant predictors in this model, but other variables were not. In the third step, CRP (in hospitalization and discharge time), SGOT, and SGPT (in discharge time) were added. Finally, this model predicted the risk of patients’ death with these variable by inter method.

Based on result, older age (OR = 0.95, *p* = 0.015) and higher length of hospitalization (OR = 0.96, *p*=0.019 0.) significantly decreased the chance of survival. Also, discharge WBC, discharge SGOT, and SGPT significantly decreased the chance of survival. Patients with a diploma level of education (OR = 0.1, *p* = 0.016) were more likely to report death than university graduates. The final model showed that the type of medicine antiviral (OR: 11.10, *p* = 0.038) than reference (antiviral and corticosteroid), and discharge O2 saturation (OR: 1.10, *p* < 0.001) had a positive association with the chance of survival, but other variables were not considered as predictive variables.

**Table 4:** Hierarchical logistic regression predicting the death of the COVID-19 patient (n = 1030)

Variables			Model1		Model2			Model3	
			OR	95% CI	OR	[95% CI]		OR	[95% CI]
Age			0.96^***^	[0.94,0.98]	0.97^*^	[0.93,1.00]		**0.95** ^*^	[0.91,0.99]
Education									
Lower than diploma			0.52	[0.15,1.84]	0.28	[0.06,1.21]		0.51	[0.10,2.66]
Diploma			0.07^***^	[0.02,0.28]	0.122^**^	[0.02,0.6]		**0.10** ^*^	[0.02,0.65]
Ref			-	-	-	-		-	-
Length of hospitalization			0.92^***^	[0.89,0.95]	0.96	[0.93, 0.99]		**0.96** ^*^	[0.93,0.99]
Underlying diseases									
Not have					1.59	[0.45,5.56]		1.77	[0.42,7.43]
Respiratory					3.14	[0.91,10.81]		3.18	[0.73,13.89]
Internal					0.98	[0.26,3.72]		0.82	[0.20,3.31]
Ref					-	-		-	-
History of covid19					0.54	[0.14,2.05]		0.25	[0.05,1.24]
Ref					-	-		-	-
Type of medicine									
Antivirus					17.60^**^	[1.96,157.73]		**11.10** ^*^	[1.14,107.89]
Corticosteroid					1.29	[0.46,3.62]		1.13	[0.32,3.99]
Ref					-	-		-	-
O2 saturation	Admition				1.04	[0.99,1.10]		1.04	[0.98,1.10]
	Discharge				1.10^***^	[1.05,1.16]		**1.1** ^***^	[1.05,1.17]
WBC	Admition				0.98	[0.88,1.09]		0.92	[0.81,1.04]
	Discharge				0.90^***^	[0.83,0.97]		**0.87** ^**^	[0.80,0.96]
CRP	Admition							0.99	[0.97,1.01]
	Discharge							0.99	[0.98,1.02]
Discharge SGOT								**0.97** ^***^	[0.95,0.98]
Discharge SGPT								**0.98** ^***^	[0.97,0.99]
Nagelkerke R Squared		**0.356**			**0.735**		**0.794**		
Predicted Percent Correct 79.2 90.893.1									

*P < 0.05, **p < 0.01, ***p < 0.001

*Note* Reference’s level of variable defined in footnote of before table

### CART method

**Fig. 1:**
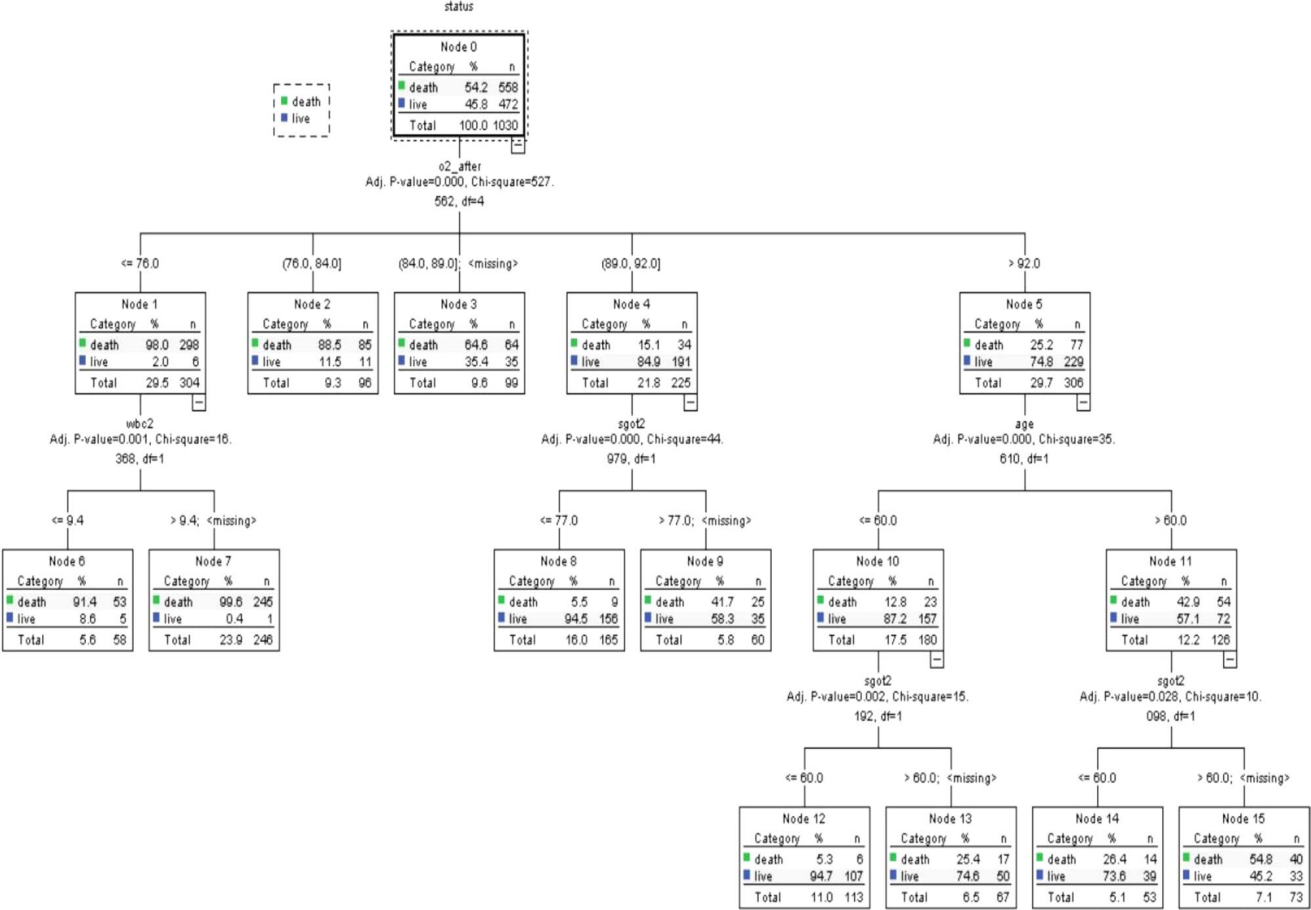
Classification tree for predicting death among covid19 patientsbased related variable

Figure 1 displays the optimal classification tree, segmented by the most influential predictive variable.

Table 5 shows the comparison of the predictive power of the different approaches in terms of the, sensitivity, specificity, and area under the ROC curve. Hierarchical logistic regression and CART models had similar performance. While in the regression model, at each step, adding variables and controlling for previous variables increases the prediction accuracy of the model. The classification model, albeit with a slight difference, has higher accuracy compared to the final regression model.

**Table 5:** Predictive performance of different multivariate models

Model		Sensitivity^*^(95%CI)	Specificity^*^(95%CI)	AUC^*^(95%CI)
Hierarchical logistic regression	Stage1	74.6(62.06–84.73)	80.42(74.82–85.24)	77.27(72.13–81.86)
	Stage2	85.14(81.96–87.95)	84.5(80.85–87.69)	84.85(82.51–86.98)
	Stage3	89.03(84.34–92.71)	78.88(73.31–83.76)	84.38(80.85–87.49)
CART(original sample)		90.24(81.68–95.69)	90.95(86.37–94.38)	90.57(86.7-93.61)
CART(validation sample)		91.95(84.12–96.7)	93.52(89.36–96.41)	92.67(89.13–95.34)

*Expressed in Percents(%)

AUC: Area under ROC Curve

Predictive models for the final outcome(death) achieved accuracies ranging from 81 to 87% for hierarchical multiple logistic regression and from 87 to 94% for the CART model. Therefore, the CART model performed better than the hirerical multiple logistic regression model. Although the CART model showed higher accuracy, the differences in AUC are not statistically significant, and the results should be interpreted with caution.

## Discussion

In this study, the relationship between the demographic, laboratory, and clinical information was investigated in patients with confirmed COVID-19. These patients were selected from those who were admitted in Ali Asghar, Nemazee, Shahid Faqihi and Shahid Chamran hospitals in Shiraz, the capital of Fars province in the south-western Iran. In the present study, no significant relationship was found between mortality and gender, which was inconsistent with the findings of the studies conducted in China and Italy, reporting that COVID-19 mortality rate was higher in men than in women [19, 20].

The Disease Control and Prevention Center in China reported a mortality rate of 2.8% and 1.7% in men and women, respectively [21].

There is also a lack of association between gender and COVID-19 mortality rate in Turkey, which may be related to the similarity of religion in this country and Iran [22, 23].

In addition, the current study showed that COVID-19 mortality rate was higher in the elderly patients, which may be due to the fact that COVID-19 mortality rate is generally higher in patients aged > 55 years [24].

In our study, the duration of hospitalization was 12 days on average, which was close to that in COVID-19 patients admitted to Ghana hospital (10–11 days). However, it was different from the length of hospitalization in patients admitted to Vietnam and China hospitals, which was 19 to 21 days on average [25, 26].

This difference in the length of hospitalization might be due to the difference in the quality of health care systems and the implemented strategies for prevention and control of COVID-19 in different countries. In our study, the patients’ WBC count during hospitalization and discharge, especially at the time of discharge, had a significant relationship with the mortality rate. In general, increased WBC count was associated with increased mortality in our study. The results of our study and that carried out in Uttar Pradesh, India, suggest that absolute neutrophil count and WBC count, which are part of the innate immune system, increased along with the severity of COVID-19 symptoms. When the neutrophil count increases, reactive oxygen species, which can damage the normal and foreign cells, are released and reduce the lymphocyte count needed to fight against infectious diseases. This factor is suitable for measuring and predicting the probability of death due to COVID-19 [27, 28].

In our study, patients with lower education level (high school diploma or lower) had a much lower chance of survival than those who had university education. This finding is probably due to the fact that patients with a lower level of education have less information about the ways to prevent disease transmission and infection, such as how to properly wash hands, strengthen the immune system, wear face mask, and adhere to quarantine principles [29].

In line with a study conducted in Turkey, we also found that patients whose SGOT, SGPT, and CRP levels were uncontrolled and higher at the time of discharge had a lower chance of survival [22].

A significant increase of SGOT, SGPT, and CRP levels can be an indicator of liver failure.

The level of these two enzymes, as an early warning sign of the disease, can help to classify the COVID-19 and pneumonia into mild, moderate, and severe categories and to decide about admission or non-admission of patients in the ICU. In the present study, with the increase of blood O2 saturation rate during hospitalization and discharge, the chances of survival of patients increased, which was in line with the findings of a study done by Oliveira et al. [30].

On the other hand, in our study, the patients who only used antiviral medications had a much better recovery than those who simultaneously took antivirals and corticosteroids. Chang et al., (2022) reported that treatment with corticosteroids was responsive to ARDS diseases other than COVID-19 [31].

Due to their anti-inflammatory and immunosuppressive properties, corticosteroids are used to relieve the symptoms induced by COVID-19.

One of our study limitations included the incompleteness of some of the patients’ medical file. An appropriate sample size (*n* = 1030) and inclusion of all COVID-19 patients in Shiraz can be the strengths of this study. Future studies are suggested to investigate the problems which occur after being infected with COVID-19.

### Electronic supplementary material

Below is the link to the electronic supplementary material.

Supplementary Material 1

## Data Availability

The datasets generated and/or analyzed during the current study are available from the corresponding author upon reasonable request.

## Abbreviations

WBC: White Blood Cells
CRP: C-Reactive Protein
SGOT: Serum Glutamic-Oxaloacetic Transaminase OR AST: Aspartate Aminotransferase
SGPT: Serum Glutamic Pyruvic Transaminase OR ALT: Alanine Aminotransferase
po2: Partial Pressure of Oxygen
CART: Classification And Regression Tree
AUC: Area under ROC Curve
