# Supplementary material for: The association between potential predictors and death of patients during the COVID-19 pandemic in Shiraz: a hierarchical multiple regression analysis
Source: BMC Public Health. 2024 Jul 23;24:1975. doi: 10.1186/s12889-024-19372-2 (PMC11267688; doi:10.1186/s12889-024-19372-2)
Supplement: Supplementary file 1 — Supplementary Material 1 [file 12889_2024_19372_MOESM1_ESM.pdf]

Questionnaire

A) Demographic Questions:

1)Gender Female ☐ Male ☐

2) Education Lower than diploma ☐ Diploma ☐ University ☐

B) Questions on Clinical and Preclinical Symptoms:

3) Does the patient have a history of underlying diseases?

Yes ☐ (Respiratory ☐ Internal☐ Internal-respiratory☐)

No☐

4) Have they had a history of hospitalization due to COVID-19? Yes☐ No☐

5) Type of medication used:

Antivirus ☐ Corticosteroid ☐ Antivirus & Corticosteroid ☐

6) Have they had a history of hospitalization due to COVID-19? Yes☐ No☐

C) Laboratory Indices:

Time of Death	Discharge Time	Admission Time	
			WBC
			CRP
			SGOT

			SGPT
			Total bilirubin
			Direct bilirubin
			Po2
			O2 **S**aturation
